# Life cycle assessment of sustainable air-cured alkali-activated concrete for permeable pavements using agro-industrial wastes

**DOI:** 10.1038/s41598-025-04783-x

**Published:** 2025-07-01

**Authors:** Shriram Marathe, Akhila Sheshadri, Vsevolod Nikolaiev

**Affiliations:** 1 Department of Materials Engineering and Construction Processes, Faculty of Civil-Engineering, Wrocław University of Science and Technology, Politechnika Wrocławska 27, 50-370 Wrocław, Poland; 2 Department of Civil-Engineering, Nitte (Deemed to Be University), NMAM Institute of Technology (NMAMIT), Udupi District, Karkala Taluk, Karnataka 574110 India

**Keywords:** Alkali activation, Permeable concrete, Embodied energy, Life cycle analysis, Carbon footprint, Agro-industrial wastes, Civil engineering, Environmental impact

## Abstract

This study evaluates the environmental performance of air-cured alkali-activated permeable concrete (PAC) developed using agro-industrial by-products, including sugarcane bagasse ash (SBA), recycled concrete aggregates (RCA), waste foundry waste sand (WFS), and ground granulated blast furnace slag (GGBS). A cradle-to-gate life cycle assessment (LCA) was conducted to quantify reductions in equivalent carbon dioxide emissions (ECO_2e_) and embodied energy (EE) relative to conventional OPC-based permeable concretes (OPCC). The results reveal that optimized PAC mixes achieve up to 57% lower EE and 77% lower ECO_2e_, confirming their environmental superiority. These improvements are attributed to the complete substitution of OPC with low-impact binders and the replacement of virgin aggregates with recycled counterparts. In addition to its environmental advantages, PAC demonstrated notable cost reductions of up to 60%, enhancing its viability for real-world infrastructure applications. The study further highlights the functional advantages of PAC including enhanced permeability, structural resilience, and suitability for stormwater management which make it an effective solution for sustainable pavement infrastructure. By integrating agro-industrial waste streams into concrete-composite production, the research advances the principles of resource-efficient engineering and circular economy. The findings establish a strong foundation for future studies on life cycle cost analysis, activator optimization, and implementation strategies, thereby promoting the adoption of PAC as a low-carbon alternative in urban infrastructural practices.

## Introduction

The challenges of climate change and associated global warming represent significant crises of our time. To address these issues, it is essential for humanity to lower *greenhouse gas* (GHG) emissions. The recent *International Energy Agency report*s-2024 reveals that, the construction and building segment alone was accountable for 21% of GHG emissions, 34% of worldwide energy stipulate, and 37% of energy and progression-narrated carbon dioxide (CO_2_) secretions^1^. The manufacture of the Ordinary Portland Cement (OPC) dependent on finite resources, such as alumino-silicate raw materials, and contributes substantial CO_2_ emissions, mainly due to the calcination of limestone. Civil engineers are continually searching for alternative materials in concrete construction that can enhance structural performance and promote sustainable resource management^2^. Therefore, there is a pressing need to explore alternative edifice materials that can be utilized in similar applications but with a reduced ecological footprint. *Alkali-activated material*s (AAMs) and *geopolymers* (GP) are promising options, as they do not require the high-temperature de-carbonization process typically associated with traditional OPC production^3^. Recent studies have advanced the understanding of AAMs by exploring a wide range of precursors and activators. For instance, *Heshmat* et al. examined the effects of activator composition and binder on the mechanical and durability traits of slag-based AAM concrete, demonstrating that tailored activator formulations can significantly enhance performance. *Žūrinskas* et al. developed sustainable binders by activating blends of iron sludge and biomass ash, revealing the potential for waste-derived materials in producing low-impact construction composites. Similarly, *Guo* et al. illustrated how mine tailings can be transformed into high-value alkali-activated concretes, supporting circular economy goals and contributing to sustainable urban development^4–6^.

These materials are viewed as potential solutions for achieving sustainable construction in the future, given their significant reductions in both *gross energy requirements* (GER) and *global warming potential* (GWP) contrasted to OPC of equivalent strength. Furthermore, the manufacture of AAMs are anticipated to contribute to waste reduction alongside mitigating global warming^7^. Moreover, have demonstrated the potential to reduce carbon footprints by approximately 9% to as much as 80% compared to conventional OPC systems. This broad range stems from variations in precursor materials (such as slag, fly ash, biomass ash), types and concentrations of alkaline activators, curing regimes, and local energy and resource profiles. As such, environmental benefits are highly context-dependent and should be evaluated using region-specific life cycle inventories^8–10^.

The *Life Cycle Assessment* (LCA) is generally identified as a valuable method for evaluating the ecological impacts and for comparing their purported environmental advantages^7^. As per ISO 14,040:2006^11^, LCA is described as “*the compilation and evaluation of the inputs, outputs, and potential environmental impacts of a product system throughout its life cycle*.” This process generally involves four primary stages: (i) defining the scope and objectives, (ii) instituting an inventory for the life cycle progressions, (iii) characterizing and measuring the life cycle impacts, and (iv) inferring the results. In distinction to the extensive studies conducted on the LCA of cementing-based products, which have been researched for many years, the LCA of AAM products is still in its early stages and requires further long-standing investigations. However, initial findings suggest that the GHG emission profiles of AAMs vary significantly depending on the choice of precursors, which includes factors such as the extraction and mining methodologies, the treatment and transportation of unprocessed products, the alkaline activating chemicals employed, and the adopted methods of curing^7^. The making of alkaline activators has a substantial ecological impact considering GWP, primarily due to the high electricity consumption associated with their manufacturing processes. Consequently, selecting different activators or utilizing alternative energy sources for electricity can significantly lower the GWP of resulting AAMs. One viable option is to incorporate by-products as alkaline-activators and rely on renewable energy resources for electricity generation. A key pronouncement reported in the literature is that while several AAM mixtures demonstrate notable reductions in global warming potential, they may also result in increased impacts in additional ecological categories, such as *Marine Eutrophication* and *Ozone Depletion*. Furthermore, the performance of AAM rammed earth and bricks showed greater assure compared to conventional clay bricks and OPC-based binders used in earth materials^7^. Urban planners must create systems that are sustainable and resilient to the impacts of climate change within city environments. For instance, implementing *Green Infrastructure* (GI) can effectively reduce runoff from roadways, manage diffuse pollution, and contribute to carbon sequestration. These advantages are crucial for addressing climate change and adapting to shifts in rainfall patterns. Additionally, GI offers a viable alternative for urban development that aligns more closely with the needs for urban adaptability, resilience, and sustainability^12^. On the other hand, climate change is raising urban temperatures, especially in heat islands. This issue is intensifying in growing cities, emphasizing the need for research on mitigation and adaptation strategies like reflective materials, green roofs, cool roofs, parks, and permeable pavements^13^. Benefits of permeable pavements include pollution control, decreased aquaplaning, reduced surface runoff and noise from vehicles, and improved qualitative parameters. Such pavements are the part of the *Sustainable Urban Drainage System* (SUDS) categorization, which aims to locally treat stormwater and mitigate the impact of impermeable locales on drainage systems. Optimizing metropolitan drainage is essential to reduce flooding, especially as heavy rainfall frequency increases. SUDSs offer a path for this optimization. But, the intricacy of GIs and SUDS systems makes it challenging to determine which technologies and designs to prioritize. The LCA is a key technique for appraise the ecological impact across all stages of life cycle. The LCA of permeable pavements is a relatively current and growing area of research study^14^

There are very limited studies on the literature which focus on the developments in the LCA of permeable concretes developed using GPs/AAMs. Huang and Wang assessed the engineering behaviors and sustainability impacts of GP permeable concrete made with metakaolin (MK) and fly ash (FA), compared to traditional OPC based PCs, and emphasized LCA to quantify energy expenditure and GHG emanations. The findings reveal that GP pervious concrete exhibits superior mechanical strength and durability, while FA-based concrete offers lower energy use and GHG emissions^15^. Recently, **Wu** et al. investigated the use of waste seashell powder (SP) as a cementing additive in fly-ash based AAC and demonstrated that AAC with 45% SP significantly lowers carbon emanations by roughly 60–70%^16^. The study by Huang and Wang evaluates the attributes of a type of GP pervious concrete utilizing recycled (RCA) and natural coarse aggregate (NCA). The LCA results revealed a lower GWP and eutrophication potential for GP mixes, despite higher energy consumption. The findings underscore the benefits of using RCA, contributing to reduced environmental impacts during material acquisition and transportation, while maintaining performance through enhanced bonding properties in alkali-activated mixtures^17^.

This study particularly investigates the development and environmental evaluation of *alkali-activated permeable concrete* (PAC) utilizing by-products (agro-industrial) and recycled materials as sustainable alternatives to conventional components. Marathe et al. developed PAC composites using agro-industrial wastes such as sugarcane bagasse ash (SBA) and cast-off aggregates from construction-demolition dissipate (RCA). GGBS serves as the primary binder, while SBA replaces a portion of the binder to enhance sustainability, and the RCA, derived from construction and demolition waste, substitutes NCA at varying proportions to reduce reliance on non-renewable resources. The study optimized mix designs for hydraulic conductivity and compressive strength, achieving optimal mechanical properties with 10% SBA and 50% RCA. Additionally, WFS is incorporated as fine aggregate^18^. The alkali-activator solution, encompassing liquid sodium silicate (LSS) and sodium hydroxide (NaOH), is optimized to achieve desired mechanical properties. Key results show that incorporating SBA enhanced compressive strength (up to 37.49 MPa) while maintaining desirable permeability (k-values around 3.50 mm/s). The mixes demonstrated balanced workability, density, and water absorption characteristics suitable for paving applications. Additionally, flexural fatigue tests revealed that SBA significantly improved fatigue life, particularly in mixtures with RCA. Statistical analyses using Weibull distribution confirmed the reliability and performance of the mixes in cyclic loading scenarios, indicating a promising avenue for sustainable pavement solutions that effectively utilize agro-industrial by-products and recycled materials^19^.

Continuing the research from the reported work^19,20^, this study’s main objectives include analyzing the GWPs of the matching PAC composites with the main focus on, embodied *carbon dioxide equivalent* (ECO_2e_) and *embodied energy* (EE) to quantify their environmental benefits over analogous permeable OPCC pavement mixes. Sensitivity analyses and LCA are conducted to identify the contributions of individual materials and to evaluate the influence of varying proportions of SBA and RCA on the overall sustainability of PAC^21^. These findings aim to advance the development of environmentally responsible and functionally effective concrete solutions for urban infrastructure applications. Also, by focusing on reductions in carbon emissions, energy consumption, and resource utilization, this research contributes to advancing sustainable permeable pavement construction practices and aligns with the principles of circular economy in pavement engineering.

## Materials, methods and mechanical performance

This section provides a concise overview of the materials and methods employed in the current study. The detailed mechanical performance data have already been extensively documented in Marathe et al.^19^. Thus, this section serves to summarize the essential aspects required to establish the context for the subsequent LCA analysis.

### Materials

This study primarily utilized ground granulated blast furnace slag (GGBS), a byproduct of industry from the iron and steel sector, augmented with the ash of sugarcane bagasse (SBA), an agro-waste, as a partial binding substitute. Coarse aggregates comprised naturally crushed stone aggregate (NCA) and recycled coarse aggregates (RCA) sourced from demolition and construction (C&D) waste. In accordance with the specifications for pervious concrete, which reduces the utilization of fine materials, the coarse-to-fine aggregate proportion was upheld at 9:1. The WFS, an additional industrial by-product, was utilized as the fine aggregate (FA). The alkaline activating solution was formulated utilizing sodium hydroxide (NaOH) granules with 98% pureness and liquefied silicate of sodium (LSS). The mixture was designed to get a modulus (Ms-value) of 1.25 and a sodium oxide dose of 4% by the total weight of the binding agent based on prior optimization for structural and longevity effectiveness for alkali-activated concrete mixes with similar mixtures^22,23^. The freshwater-to-binder (w/b) ratio was first set at 0.20 and later raised to 0.40 for the manufacture of the mix using tap water^19^.

### Mix design strategies

The mix proportion for PAC was developed for low-slump concrete having target strength of 20 MPa^24^. Initially, the mix was tailored to create permeable alkali-activated concrete (PAC) with GGBS as the primary binder and incremental replacements with SBA at levels of 0%, 5%, 10%, 15%, and 20%. The final mix proportions incorporated 290 kg of total binder per m^3^, a w/b of 0.40, and alkali activators premeditated to sustain a percolation pace exceeding 180 mm/min (Darcy’s permeability coefficient of 3.0 mm/s). RCA replacements were tested at levels of 0%, 25%, 50%, 75%, and 100% of NCA, resulting in 13 unique mix designs. Mix IDs such as “PAC-10–50” denote 10% SBA and 50% RCA replacements. Mechanical properties were evaluated using strength and hydraulic conductivity performances^19^. The Table 1 summarizes the mix proportions for 1 m^3^ of selected PAC, while Table 2 provides results of mechanical tests of each mixes^19^.

**Table 1:** Mix proportion of selected PAC mixes for producing 1 m^3^ of concrete in kg^19^.

Mix ID	Aggregates			Alkaline activator			Binder	
	FA	NCA	RCA	Water	LSS	NaOH	SBA	GGBS
PAC-0–0	199.7	1881.3	0	92.791	44.207	6.583	0	290
PAC −0–50	199.7	940.68	888.03	92.791	44.207	6.583	0	290
PAC-15–0	198.6	1871.65	0	92.791	44.207	6.583	43.5	246.5
PAC −15–50	198.6	935.83	883.45	92.791	44.207	6.583	43.5	246.5

**Table 2:** The workability and the other engineering properties of selected PAC mixes at 28 days of air-curing^19^.

Mix ID	CFV	HDD	SWA	CoP (k)	CS	STS	SFS
Unit	-	kg/m^3^	(%)	mm/s	MPa	MPa	MPa
PAC-0–0	0.78	1897	5.97	3.474	32.2	2.14	3.92
PAC −0–50	0.73	1702	7.12	4.968	26.22	1.84	3.13
PAC-15–0	0.84	1898	5.93	4.392	37.49	2.47	4.1
PAC −15–50	0.76	1792	6.51	5.096	30.1	2.05	3.51

Four-Point Static Flexural Strength (SFS); Hardened Dry Density (HDD); Compaction Factor Value (CFV); Hydraulic Co-efficient of Permeability (CoP); Saturated Water Absorption (SWA); Split Tensile Strength (STS); Compressive Strength (CS).

### Key results of mechanical properties

The mechanical performance results demonstrated satisfactory strength and hydraulic conductivity for four selected mixes: PAC-0–0, PAC-0–50, PAC-15–0, and PAC-15–50 (as accessible in Table 2). These mixes were chosen to investigate the isolated effects of SBA incorporation (PAC-15–0), RCA inclusion (PAC-0–50), and their combined influence (PAC-15–50) in comparison to the reference mix PAC-0–0. Detailed mechanical performance data have been previously reported^19^, allowing this study to focus on the environmental implications of these designs through LCA analysis. The subsequent section delves into the LCA results, offering a comparative evaluation of these PAC mixes against conventional permeable OPCC mixes.

### Life cycle analysis (LCA)

This current investigation employs an exploratory LCA to evaluate and compare the ecological impacts correlated with the making of conventional OPC-based permeable concrete (i.e., OPCC) and alternative alkaline-activated permeable composite (i.e., PAC) mixes incorporating agro-industrial wastes. The analysis specifically focuses on two key environmental indicators: equivalent carbon dioxide emissions (ECO_2e_) and embodied energy (EE), which serves as metrics to assess the environmental footprint and sustainability potential of PAC in contrast to traditional OPCC. The scope of the LCA trails a *cradle-to-gate* approach, encompassing all relevant stages from raw material extraction, processing (including energy-intensive steps such as grinding and milling), to transportation and concrete production. The assessment framework conforms to the standards defined by ISO 14,040 (Sect. 5.2) for life cycle inventory analysis. To provide a structured analysis, this section is divided into subsections. These include: (i) a detailed methodology explaining the calculation of EE and ECO_₂e_ for selected PAC and OPCC mixes; and (ii) a material-specific impact analysis identifying the dominant contributors to the total environmental load for each mix under consideration^21^.

### Brief methodology of LCA

Previous studies on concrete LCA often assume that supplementary cementing materials (SCMs) contribute negligible energy input due to their classification as industrial by-products (Assi et al.^25^). However, such assumptions may underestimate actual impacts, as they typically omit energy demands associated with collection, processing, grinding, and particle size reduction. To ensure analytical rigor, the present study incorporates these energy expenditures for all materials, thereby facilitating a more equitable comparison between OPCC and PAC systems. The adopted methodology is grounded in established LCA protocols and draws upon validated literature sources, including the works of Hammond et al.^26^, McLellan et al.^27^, and, Sobuz et al.^28^. The core objective of the analysis is to compute EE and ECO_2e_ per 1 m^3^ of pervious concretes for both conventional and alkali-activated variants. The LCA boundaries include raw material acquisition, mechanical processing, transportation, and concrete batching excluding use-phase and end-of-life considerations, in accordance with *cradle-to-gate* conventions. Ecological impact values for each material (in terms of MJ/kg and kg CO_2e_/kg) were extracted from credible databases and literature sources (see Table 3), including Thannimalay et al.^29^, Hammond et al.^26^, Bhardwaj & Kumar^30^ and Sobuz et al.^28^. Material quantities were derived from the concrete mix designs presented in Table 1, encompassing four PAC variants (PAC-0–0, PAC-0–50, PAC-15–0, and PAC-15–50) as well as the baseline OPCC mix, which consists of OPC, GGBS, NCA, river sand, and water^21^. The EE and ECO₂e for each mix were calculated by multiplying the respective material quantities by their corresponding impact values. A further breakdown of contributions by individual materials was performed to identify key sources of environmental burden in each mix configuration.

**Table 3:** Personage values of ingredient materials/kg adopted in the estimation of LCA.

Material	Aggregates			Alkaline activator			Binder		
	FA	NCA	RCA	Water	LSS	NaOH	SBA	GGBS	OPC
EE (MJ/kg)	0.081	0.083	0.021	0.20	5.37	3.50	0.036	1.60	5.50
ECO_2e_/kg	0.051	0.0052	0.004	0.0008	0.78	0.6329	0.0028	0.083	0.95
Reference	^26^	^26^	^31^	^30^	^27^	^29^	^28^	^26^	^26^

## Results of LCA

### Results of the analysis concerning to embodied energy (EE)

**Fig. 1:**
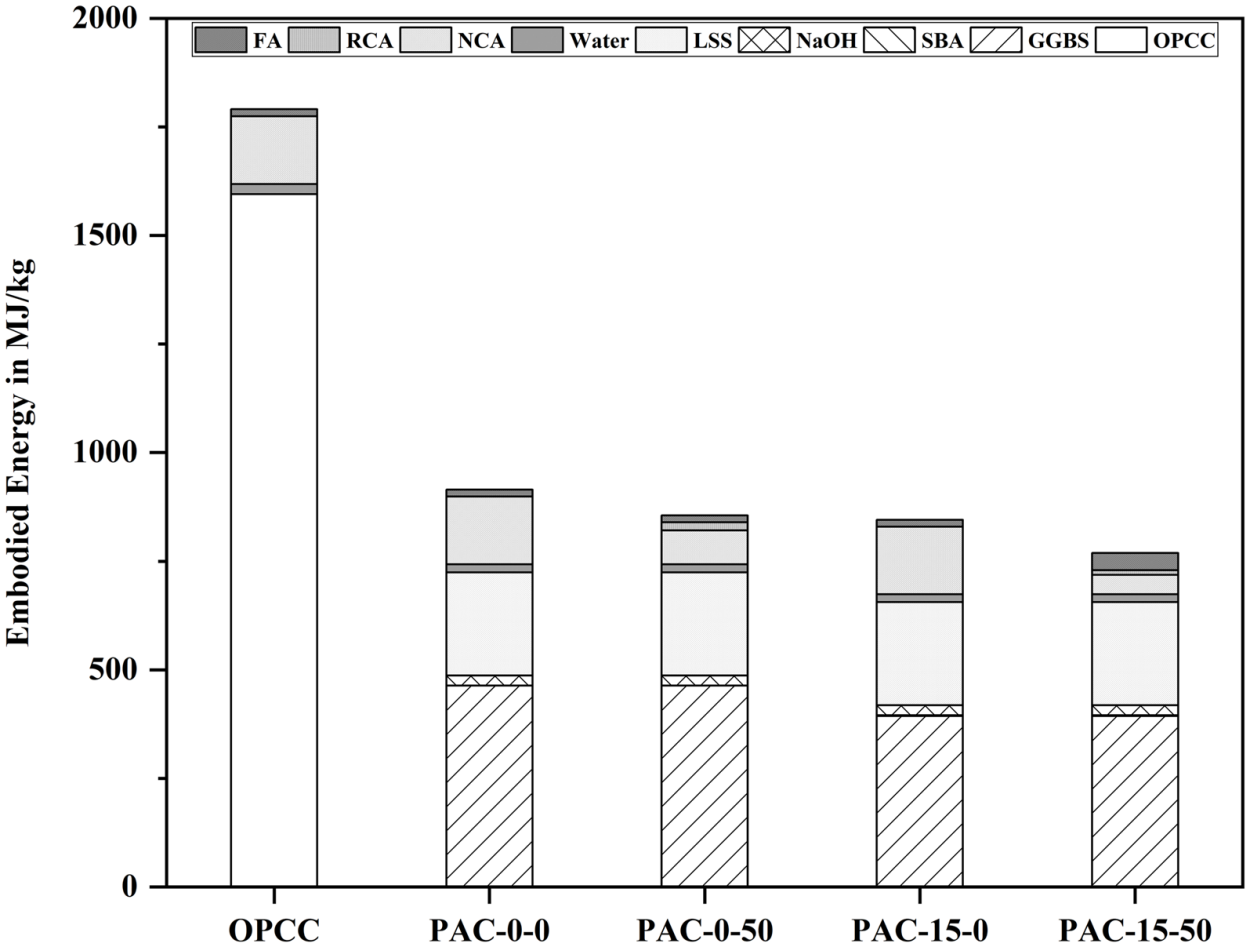
Ingredients’ involvement to the EE of permeable concrete mixes.

The EE refers to the overall energy consumed in producing and transporting the materials utilized in the concretes. The EE examination results for the 4 chosen permeable composite mixes are depicted in Fig. 1, with detailed information recapitulated in Table 3.

**Fig. 2:**
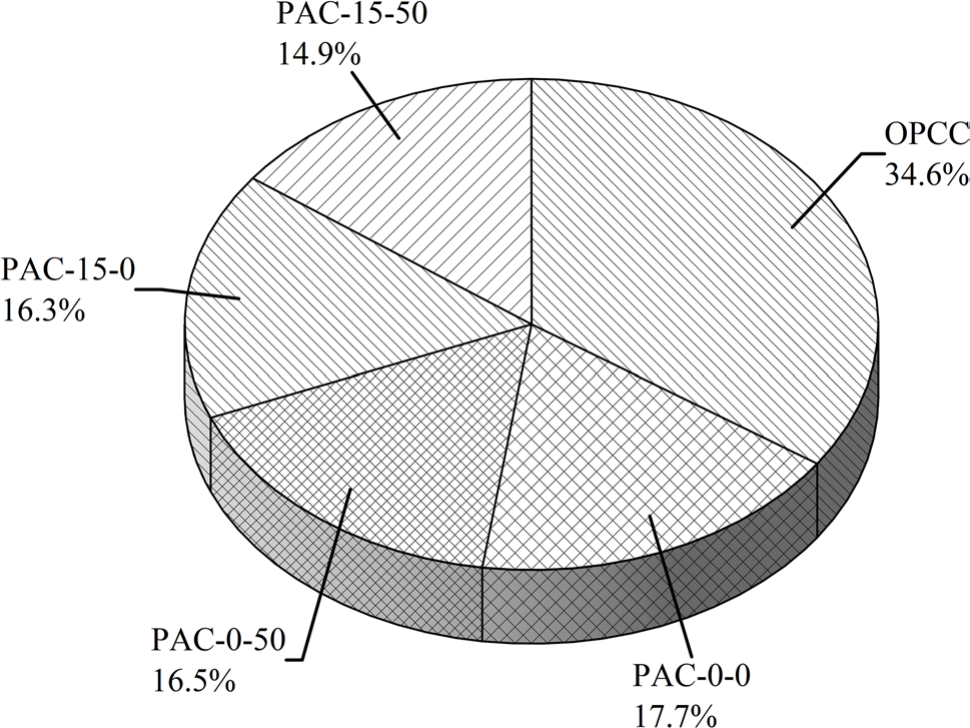
Comparing the EE of individual permeable concrete mixes.

The results indicate a substantial decrease in consumption of energy for PAC blends in comparison to OPCC alternatives. The EE of the OPCC blend was determined to be 1790.5 MJ/m^3^, while the EE of PAC-0–0 declined to 915.3 MJ/m^3^, indicating a 48.9% reduction. The PAC-0–50 mix demonstrated a reduction of 855.9 MJ/m^3^, reflecting a 52% decline. In contrast, the PAC-15–0 mix revealed an EE of 845.8 MJ/m^3^, whereas a most significant drop was noted in PAC-15–50, exhibiting an EE of 769.3 MJ/m^3^, showing a 57% decrease compared to the OPCC mix. This significant decrease is mostly due to the whole replacement of OPC with AAC precursors in the PAC mixtures. The elevated EE of OPC arises from its energy-intensive manufacturing process, whereas GGBS and SBA necessitate significantly less energy. Figure 2 further delineates the share of each ingredient in the permeable mixtures to the overall EE. In the OPCC mix, OPC accounted for 34.6% of the total EE, but in PAC-15–50, its contribution decreased to roughly 14.9%. Notwithstanding this enhancement, substances such as NaOH and LSS substantially influenced the EE of AAC mixtures. The analysis highlights the critical importance of material selection in minimizing the energy expenditure of porous composites. Substituting OPC with agro-industrial waste products (such as GGBS, WFS, and SBA) not only reduces expenditures of energy but also enhances the valorization of abandoned materials, providing both the environment and financier benefits^21^.

### Results of the equivalent carbon dioxide emissions analysis

**Fig. 3:**
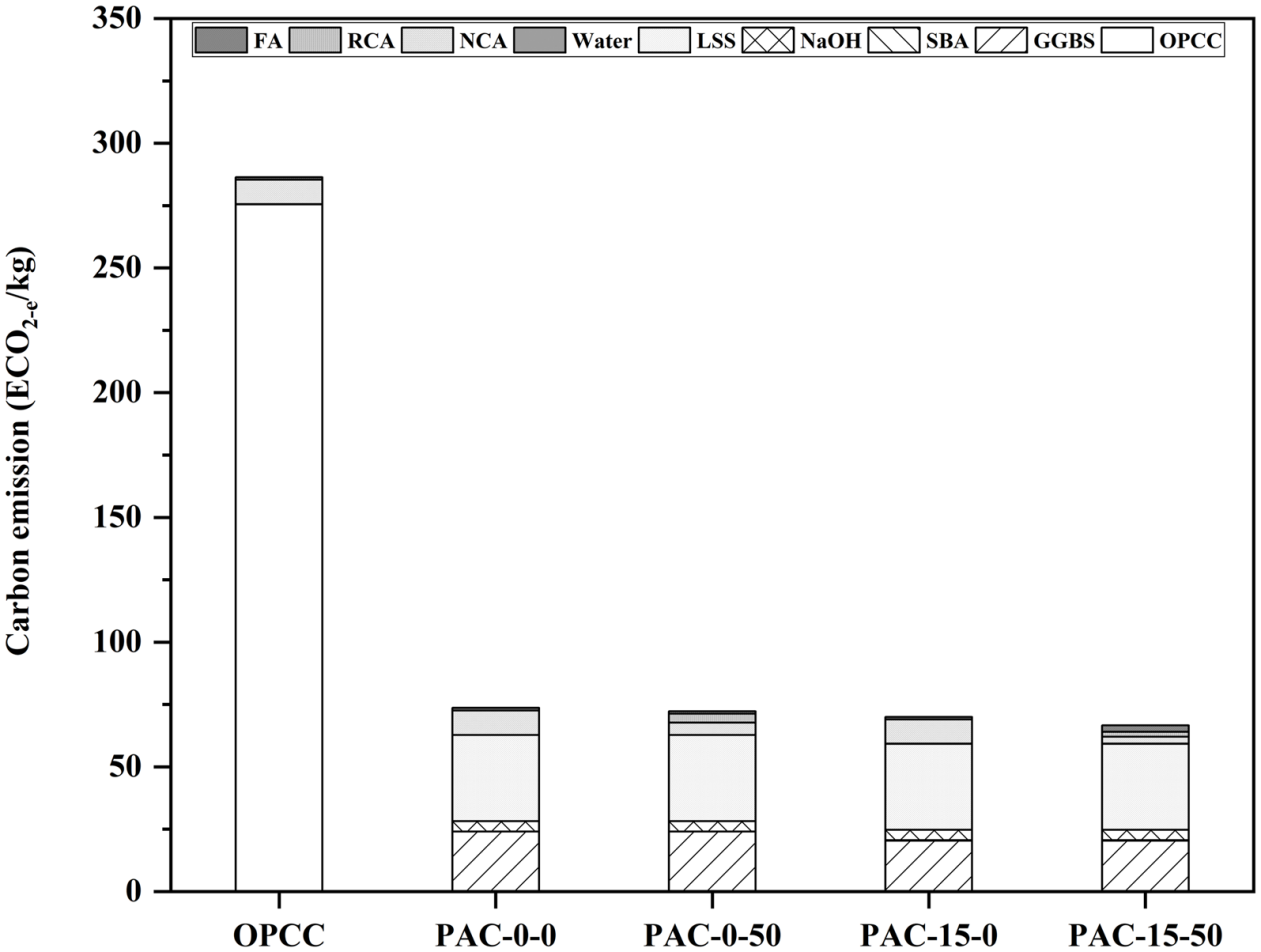
Ingredients’ involvement to the ECO_2e_ of permeable concrete mixes.

The ECO_2e_ quantify the total GHG emanations linked to the manufacturing of constituent materials, articulated in CO_2_ correspondents. In accordance with the results of the EE examination, the ECO_2e_ scores for the trial mixtures were computed and are depicted in Fig. 3. The OPCC displayed the principal CO_2_ emanations, with a gross ECO_2e_ of 286.39 kg/m^3^. Conversely, the PAC exhibited notable diminutions in such emanations. Particularly, the PAC-0–0 mix recorded an ECO_2e_ of 73.59 kg/m^3^, indicating about 74% reduction compared to the OPCC mix. PAC-0–50 demonstrated a further decrease to 72.25 kg/m^3^, representing a 75% reduction, and similarly, PAC-15–0 representing 76% reduction. The most substantial reduction was observed in the PAC-15–50 mix, with an ECO_2e_ of 66.59 kg/m^3^, marking a 77% decrease relative to the OPCC baseline. These significant reductions in carbon emissions can be attributed primarily to the exclusion of OPC, which is recognized as one of the chief carbon-exhaustive construction materials^21^.

**Fig. 4:**
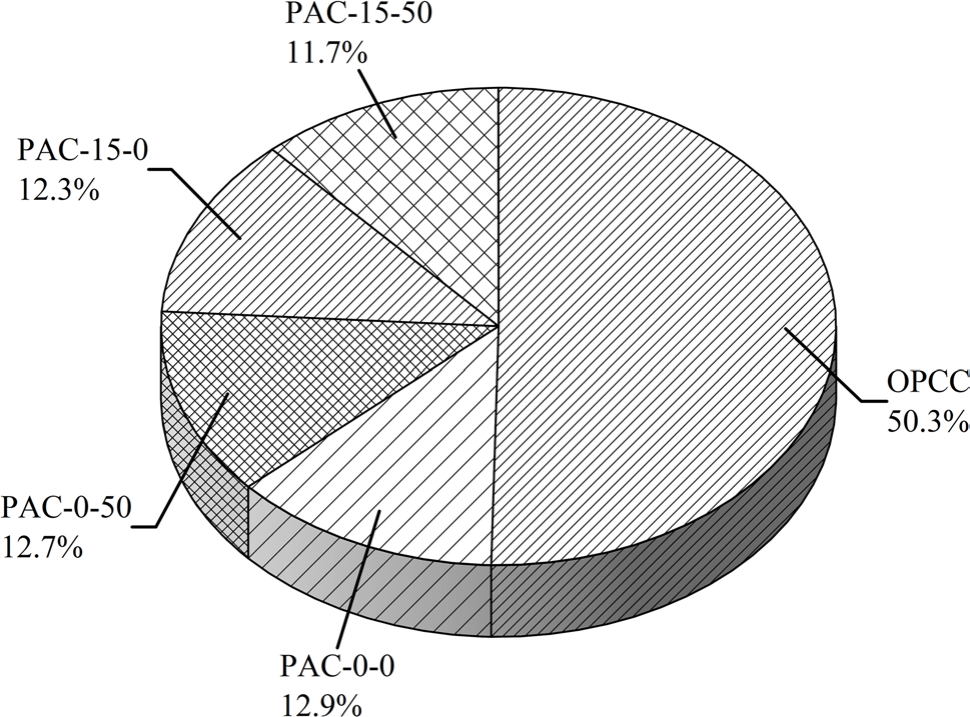
Comparing the ECO_2e_ of individual permeable concrete mixes.

Figure 4 highlights the contributions of individual materials to the gross ECO_2e_. In the OPCC, OPC alone accounted for 50.3% of the total ECO_2e_. In permeable AAC blends, the ECO_2e_ involvements were more uniformly dispersed across NaOH, GGBS, and LSS. Among these, the PAC-15–50 mix showed the lowest contribution, accounting for approximately 11.7% of the total ECO_2e_ across all permeable composite mixes. While LSS and NaOH contribute to equivalent carbon in such mixes, their blow remains appreciably subordinate than to the OPC. Overall, the results underscore the environmental advantages of AAC mixes in reducing carbon emissions. These results also proven that by reinstating OPC with by-products (agro-industrial), the AAC mixes not only mitigate GHG emissions but also provide a more sustainable alternative^21^ to conventional OPC binder based permeable concrete.

### The material contribution to the ecological impact via life cycle assessment

**Fig. 5:**
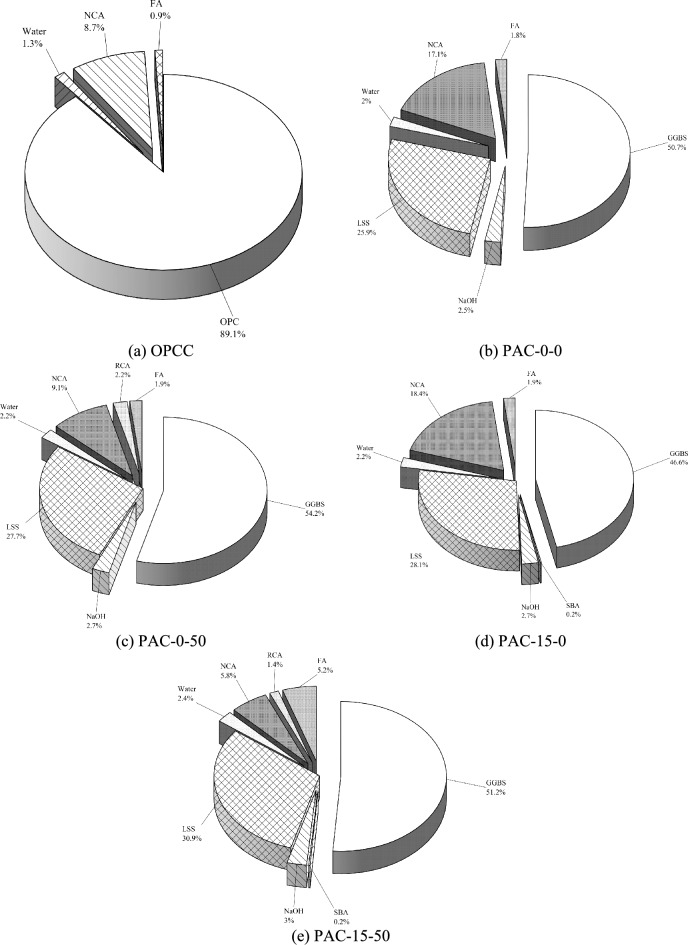
Analysis for the consideration of embodied energy in permeable concrete mixes.

**Fig. 6:**
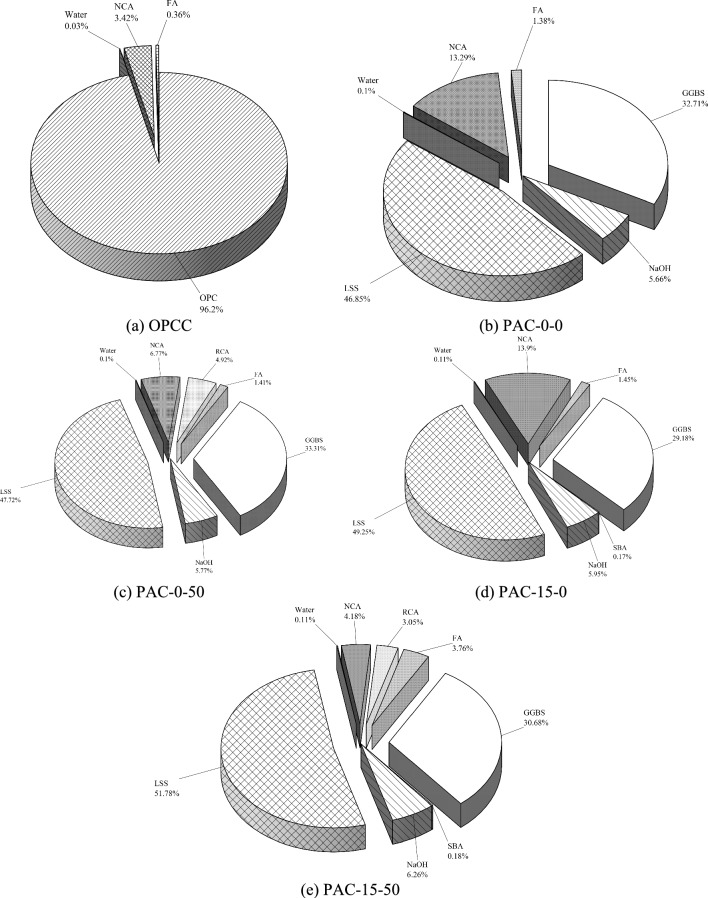
Analysis for equivalent carbon dioxide emissions in permeable concrete mixes.

The Figs. 5 and 6 respectively illustrate the relative contributions of individual materials to the total EE and ECO_2e_ in the permeable mixes. For the OPCC mix, the investigations confirm that the OPC overwhelmingly controls the environmental impact, accounting for 89.1% of the gross EE and 96.2% of ECO_2e_. These consequences underscore the critical need to curtail OPC usage to effectively diminish both energy consumption and GWG emissions in permeable pavement concrete manufacture. In distinction, the PAC mixes demonstrate a redistribution of environmental impacts, with LSS and GGBS emerging as the principal contributors to ECO_2e_ and EE. For instance, in the PAC-50–15 mix, sodium silicate is responsible for 51.78% of ECO_2e_ and30.9% of the total EE, whereas GGBS contributes 30.68% of the ECO_2e_ and 51.2% of the EE. While permeable AAC mixes significantly reduce environmental impacts compared to OPCC counterparts, these findings emphasize the importance of optimizing the production and sourcing processes of GGBS and LSS to enhance the sustainability in AACs^21^. Targeted strategies to lower the energy and carbon footprint of these materials can further improve the environmental benefits as a eco-friendly substitute to conformist permeable pavement composites.

## Overall discussions and interpretations for sustainability

Overall, the LCA of PAC incorporating agro-industrial wastes reveals significant reductions in EE and ECO_2e_, demonstrating its superior environmental performance compared to permeable OPCC counterparts. Calculations based on constituent materials and their respective EE and ECO2e values illustrate that PAC achieves reductions of approximately 50% in EE and 75% in ECO2e relative to OPCC of comparable strength grade. These findings align with studies by Zhong et al.^32^ and Singh et al.^33^, which highlight the environmental advantages of utilizing pervious concrete with alternative materials. Specific PAC formulations such as PAC-0–50, PAC-15–0, and PAC-15–50 exhibit reductions in EE ranging from 52.19% to 57.03%, while corresponding ECO_2e_ reductions vary between 74.3% and 76.74%^34–36^.

Most importantly, incorporating RCA and SBA into PAC formulations is pivotal for enhancing sustainability in concrete production. RCA replaces natural coarse aggregates (i.e., NCA), reducing dependency on non-renewable resources while diverting construction and demolition waste from landfills^37^. Simultaneously, SBA, derived from agro-industrial processes, minimizes greenhouse gas emissions and EE during its production. The PAC mixtures such as PAC-15–50 achieve up to 60% reduction in EE and approximately 75% in ECO_2e_ compared to OPCC, underscoring the transformative potential of these materials in reducing environmental footprints^38,39^. The results clearly indicate that the adoption of PAC aligns with circular economy standards by endorsing resource conservation and waste valorization. Utilizing RCA, which constitutes 60–80% of the concrete matrix by volume, significantly reduces the extraction of virgin materials and supports sustainable infrastructure development^40^. Incorporating SBA, GGBS, and waste foundry sand further enhances this alignment, optimizing resource efficiency while minimizing waste generation. By integrating these materials, PAC formulations contribute to broader sustainability objectives, as demonstrated in the reduced reliance on high-energy materials such as OPC^41^.

Moreover, the PAC’s reduced carbon footprint and enhanced resource efficiency present it as a viable alternative to OPCC for infrastructure applications. Its permeability addresses urban challenges such as storm water management and groundwater recharge, offering additional ecological benefits^42^. Moreover, the material’s durability, characterized by confrontation to chemical attacks, shrinkage, and ecological stressors, ensures long-term performance in high-demand applications such as highways and runways^43^. This reinforces the suitability of PAC for sustainable infrastructure projects, as recognized by global engineering and governmental bodies prioritizing environmentally responsible construction materials^44^. In addition to its demonstrated environmental advantages, a preliminary cost analysis further reinforces the feasibility of PAC as a sustainable alternative to OPCC. The results indicate that PAC mixes incorporating agro-industrial wastes and recycled aggregates, such as PAC-15-50 and PAC-0-50, exhibit a considerable reduction in material cost up to 60% lower than conventional OPCC per cubic meter. For instance, the PAC-15-50 mix incurs an estimated cost of INR 1967 /m^3^ compared to INR 4989 /m^3^ for OPCC, illustrating substantial economic benefits alongside ecological performance. This cost-effectiveness is primarily attributed to the replacement of energy- and resource-intensive materials (e.g., OPC and natural aggregates) with low-cost industrial by-products such as SBA, RCA, and WFS. These findings suggest that PAC not only reduces embodied environmental burdens but also offers a financially viable solution for large-scale implementation in sustainable infrastructure projects, making it an economically attractive option for municipalities and contractors seeking to balance budget constraints with green construction mandates.

Therefore, the presented LCA results clearly underscore the environmental and functional benefits of PAC incorporating RCA and SBA, which align with sustainability goals and circular economy strategies. By achieving substantial reductions in EE and ECO_2e_, PAC exemplifies an innovative solution for modern infrastructure demands, offering a balance between environmental stewardship and structural integrity. This positions PAC as a leading candidate for sustainable urban construction practices, particularly in pavement engineering applications where ecological considerations are paramount^45,46^.

## Conclusions and future directions

This study assessed the environmental sustainability of air-cured PAC mixes incorporating agro-industrial wastes specifically RCA, SBA, WFS, and GGBS through a detailed LCA. The analysis focused on EE and ECO_2e_, in comparison to conventional OPC-based permeable concretes mixes of equivalent strength. The results demonstrate that PAC formulations achieved up to 57% lower EE and 77% lower ECO_2e_, confirming their enhanced environmental performance. In addition to environmental benefits, preliminary cost analysis revealed that PAC formulations especially PAC-15-50 can be produced at significantly lower cost per cubic meter compared to OPCC, suggesting a compelling economic case for sustainable adoption. The study validated the initial hypothesis that replacing OPC with low-impact binders and using recycled materials would considerably reduce the environmental footprint of permeable pavement concrete without compromising performance. The findings also highlight PAC’s functional advantages in infrastructure applications, particularly in addressing urban challenges such as resource conservation and stormwater management. To further improve the environmental profile and practical deployment of PAC systems, the following areas are recommended for future investigation:

- Optimization of alkaline activators by exploring low-energy, renewable, or bio-derived alternatives to NaOH and LSS to significantly reduce the EE and ECO_2e_.
- Comprehensive life cycle cost analysis (*LCCA*) to assess the financially viablity of PAC in real-world infrastructure projects.
- Development of technical standards and implementation frameworks, in collaboration with industry and regulatory bodies.
- Integration with complementary sustainable technologies, such as carbon capture additives or multifunctional construction systems.

Overall, PAC offers a viable pathway toward circular economy-based, low-carbon urban infrastructure. Continued research and standardization can facilitate its transition from research to widespread application in sustainable pavement engineering.

## Data Availability

Data generated will be made available on reasonable request to the corresponding author.

## Declarations

### Gen-AI usage

In preparing this manuscript, the authors utilized the *Voilà AI* service to evaluate the language clarity and readability of select sections. Following this, all content was meticulously reviewed, revised, and refined by the authors to ensure its accuracy and alignment with the intended scholarly standards. The authors assume full responsibility for the integrity and authenticity of the work presented in this publication.
